# Response of height, dry matter accumulation and partitioning of oat (*Avena sativa* L.) to planting density and nitrogen in Horqin Sandy Land

**DOI:** 10.1038/s41598-019-44501-y

**Published:** 2019-05-28

**Authors:** Kai Gao, Yang Fu Yu, Zhu Tie Xia, Gao Yang, Zhao Li Xing, Li Tian Qi, Lin Zhi Ling

**Affiliations:** 1 College of Agronomy, Inner Mongolia University for Nationalities, Tongliao, 028043 China; 2 Inner Mongolia Autonomous Region Feed Crop Engineering Technology Research Center, Tongliao, 028000 China; 3 Institute of Grassland, China Agricultural University, Beijing, 100193 China

**Keywords:** Plant sciences, Genetics

## Abstract

Nitrogen fertilizer application and planting densities were key limiting factor for the physiological metabolism and growth of *Avena sativa* L. In the paper, it was set five nitrogen fertilizer application (including: N1:225 kg/hm^2^, N2:300 kg/hm^2^, N3:375 kg/hm^2^, N4:450 kg/hm^2^, N5:525 kg/hm^2^) and four planting densities (including M1:3 million plants/hm^2^, M2:3.5 million plants/hm^2^, M3:4 million plants/hm^2^, M4:4.5 million plants/hm^2^), and measure plant height, aboveground biomass, underground biomass, total biomass, stem biomass, leaf biomass, root biomass and ear biomass, and calculate stem/leaf ratio, root/crown ratio, stem contribution rate, leaf contribution rate, root contribution rate and spike contribution rate, to study the effect of density and nitrogen fertilizer on height, biomass and material distribution of *Avena sativa* L to provide scientific basis for water and fertilizer management of *Avena sativa* L in Horqin Sandy Land (Science and Technology Park of Inner Mongolia university for nationalities). The results showed, The main purpose was to harvest aboveground biomass such as oat stem and leaf, the optimal density and nitrogen fertilizer combination was M4 and N3, and aboveground biomass was 4254.96 g/m^2^ (2017) and 4226.21 g/m^2^ (2018). Under the same planting density, the height, aboveground biomass and underground biomass of *Avena sativa* L all showed first increased and then decreased with the rising of fertilizer application and the biggest height value appeared at N3. Under the same nitrogen fertilizer application, the height indicated increased gradually with the rising of density, and higher biomass was obtained under lower nitrogen fertilizer application and higher planting density conditions (such as:N1 conditions, the biggest value of underground biomass and total biomass was obtained under M1 condition), and under higher nitrogen fertilizer application conditions the higher value of biomass was obtained under lower planting density condition (M1 (2017) and M2 (2018) under the condition of N5 treatment, the aboveground biomass and total biomass were the highest, while M4 (2017) and M3 (2018) underground biomass was the highest.). Under same nitrogen fertilizer application conditions, the root/shoot ratio and stem/leaf ratio all showed increased first and then decreased with the rising of density. Under same density conditions, the root/shoot ratio and stem/leaf ratio indicated different variation laws with different nitrogen fertilizer application amount. The changes laws of root contribution rate, stem contribution rate, leaf contribution rate and ear contribution rate were different due to density and nitrogen fertilizer application.

## Introduction

*Avena sativa* L. is one of the most important forage crops in the development of animal husbandry. And it was annual herbaceous plant with strong adaptability, wide distribution, high yield, good quality and easy cultivation. Its seeds are good quality materials for cattle, sheep and horses, and the stems and leaves were easy to silage and make hay. It was an important forage fodder for winter and spring supplementation, which plays a very important role in stabilizing livestock production^1–3^.

Nitrogen was a key limiting factor for the physiological metabolism and growth of crops^4^. Nitrogen fertilizer plays an extremely important role in improving the yield per unit area of food crops. And nitrogen fertilizer application amount accounts for about 2/3 of total fertilizer application amount, but utilization rate is less than 40%. Excessive application of nitrogen fertilizer not only aggravates greenhouse gas emissions from ammonia volatilization and denitrification, but also aggravates eutrophication due to nitrogen leaching^5–7^. For panting density was an important factor for crop yield, and it was one of important way to improve crop yield by appropriately increasing planting density^8^. However, the competition of crops on water, light, fertilizer and other resources become more intense with the increasing of planting density and resulting in the plant stem becoming thin and the root growth being restrained, rising intraspecific competition and the risk of lodging^9^. It was important influence factors on oat production for nitrogen fertilizer and planting densities, and some studies have showed that there was significantly influence of nitrogen fertilizer and planting densities on oat hay yield, such as reproductive allocation, effective tiller number and spikelet number etc^10,11^. Therefore, studies on nitrogen fertilizer and density of oat have important significance for improving oat yield and quality.

There were a lot of research on Avena sativa L. on domestic and foreign scholars, such as how to improve the yield and quality seeds, silage and hay oat vegetation modulation, variety breeding, high-yield cultivation techniques etc.^10–14^. And the density and nitrogen fertilizer are the main factors to limit the yield of Avena sativa L., which was hot question too. For nitrogen fertilizer, which was an important factor limiting yield and quality of Avena sativa L. But large amount of nitrogen fertilizer, which will lead to grow rapidly in stem, and diameter of stem thinner and easy to lodging, and thus affect the harvest Avena sativa L and hay quality; Insufficient application of nitrogen fertilizer will slow the growth of Avena sativa L, decrease in leaf area index, decrease in light and efficiency, and decrease in yield. For density, it mainly Avena sativa L yield by the root nutrition competition and leaf density light energy use efficiency, unreasonable planting density can cause light energy using efficiency drop, the low level of production. High density, inhibition of Avena sativa L tillering, thereby reducing stem diameter and production.

In China, *Avena sativa* L. was mainly planted in alpine pastoral areas, such as Tibet, Qinghai, Gansu, Ningxia etc, and which was as one of the important measures to solve the problem of high efficiency production and sustainable development of animal husbandry in the region^15^. The study area were mainly concentrated in the region, and there was relatively few about related research of Avena sativa L in Horqin sandy Land, and the main research content concentrated variety selection, nitrogen, spacing and suitable seeding rate ect. And there was few study on the response of dry matter accumulation and distribution of *Avena sativa* L. on planting density and nitrogen addition. So the article discuss the effect of planting density and nitrogen addition on biomass, material distribution, stem/leaf ration and root/shoot and so on in Horqin sandy land to provide theoretical basis and technical support.

## Materials and Methods

### The natural conditions of the experiment

The experiment was conducted at the Inner Mongolia University for the Nationalities experimental field station in Tongliao (Inner Mongolia, China). It is a semi-arid region with a temperate monsoon climate. The annual precipitation is approximately 399 mm, with 50–60% of total precipitation falling in August and September. The mean annual temperature is 6.4 °C. The accumulated temperature (>10 °C) is 3184 °C. The frost-free period is approximately 150 d, from May to September. The soil is gray meadow, with a pH 8.2 (pH water), organic matter content is 18 g/kg, and available nitrogen, phosphorus, and potassium is 62, 39 and 185 mg/kg, respectively. Rainfall and temperatures during the experimental period.

### Experiment design

*Avena sativa* L. was planted on April 20, 2017 and April 25, 2018, and depth 3 cm. Five nitrogen fertilizer application gradients were set, including N1:225 kg/hm^2^, N2:300 kg/hm^2^, N3:375 kg/hm^2^, N4:450 kg/hm^2^, N5:525 kg/hm^2^, and four planting densities: M1:3 million plants/hm^2^, M2:3.5 million plants/hm^2^, M3:4 million plants/hm^2^, M4:4.5 million plants/hm^2^. The plot area was 10 m^2^, 5 repeats, and the plot was randomly arranged.

### Determination method

Height determination: 10 plants were randomly selected to determine the vertical height in each community on July 15, 2017 and July 15, 2017.

Biomass measurement: on July 15, 2017 and 2018, in every community take 1 m^2^ as a sample, the ground biomass, the ground within the 1 m^2^ mowing, at the same time, the range of 0 to 50 cm depth oat root dug out, cleaning, the surface water with blotting paper net, weigh and fresh weight.

Root, stem, leaf and spike biomass measurement: 10 strains were randomly selected in each district, the whole plant (including root, stem, leaf and spike) excavated in organ separation, drying, the determination of each organ biomass, and used to calculate the related indicators.

### Data analysis

#### Relevant calculation

Total biomass = terrestrial biomass + root biomass.

Root ratio = root biomass/total biomass.

Stem/leaf ratio = stem/leaf weight.

Single plant total weight = root weight + stem weight + leaf weight + ear weight.

The contribution rate of stem is equal to the weight of the stem and the total weight of the single plant.

Blade contribution = blade weight/single plant weight.

The root contribution rate = root weight/single plant weight.

Ear contribution rate = ear weight/single plant total weight.

#### Data analysis

DPS14.0 was used for single factor analysis and correlation analysis.

## Results

### The effect of planting densities and nitrogen fertilizer on height of *Avena sativa* L

**Figure 1:**
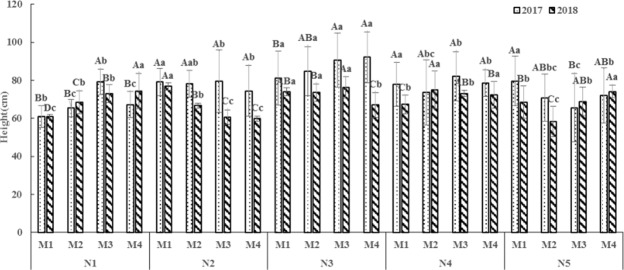
The effect of density and nitrogen fertilizer on height of *Avena stativa* L. Note: The capital letters indicated there was significantly difference at 0.05 levels under different planting density and same nitrogen fertilizer application, and lowercase letters indicated there was significantly difference at 0.05 levels under different nitrogen fertilizer and same planting density. The same below.

The height of oat showed first increased and then decreased with rising of nitrogen fertilizer under the same densities and different nitrogen fertilizer condition in 2017, and the biggest value was appeared under N3 conditions. In 2018, the change law of height was similar with 2017 except for the M4 planting density, and the height of N1 and N5 was significantly higher than N2, N3 and N4, N2 and N3 remarkable higher than N3. The height of oat indicated a trend of increasing with the rising of densities under the same nitrogen fertilizer and different densities conditions (Fig. 1).

### The effect of planting densities and nitrogen fertilizer on biomass of Avena sativa L

Under the same densities and different nitrogen fertilizer, the biomass (including aboveground biomass, underground biomass and total biomass) appeared different changing trend. Such as aboveground biomass and total biomass was the highest value under M1 condition, and underground biomass of M3 (2017) and M4 (2018) under N1 conditions. And under N2 condition, aboveground biomass and total biomass of M4 were the highest, and underground biomass of M3 was the highest. And under N3 condition, the highest value of underground biomass, aboveground biomass and total biomass was M3 in 2017 and 2018. And under N4 condition, the highest value of aboveground biomass and total biomass appeared in M1 (2017) and M3 (2018), underground biomass of M2 (2014) and M3 (2018) was the biggest. And under N5 condition, aboveground biomass and total biomass appeared in M (2017) and M2 (2018), underground biomass of M4 (2017) and M (2018) was the highest. Aboveground biomass and underground biomass all showed first increasing and then decreasing with rising of nitrogen fertilizer under the same densities, and the highest value appeared in N2, N3 and N4 due to different densities (Table 1).

**Table 1:** The effect of densities and nitrogen fertilizer on biomass of oat (g/m^2^).

		Aboveground biomass		Underground biomass		Total biomass	
		2017	2018	2017	2018	2017	2018
N1	M1	3050.52 ± 205.12Ac	3161.26 ± 121.81Ac	341.24 ± 64.26Bab	483.29 ± 37.77Ba	3391.74 ± 165.83Ac	3644.55 ± 152.23Ac
	M2	2891.56 ± 120.22ABbc	2527.35 ± 157.49Cc	364.14 ± 45.04Bb	521.53 ± 49.15Bb	3255.70 ± 150.53Ab	3048.88 ± 205.75Bc
	M3	2644.96 ± 503.60ABb	2936.09 ± 136.31ABc	599.70 ± 94.12Aa	576.54 ± 74.47Ab	3244.66 ± 345.85Aab	3512.63 ± 134.27ABd
	M4	2213.68 ± 202.44Bc	2768.42 ± 193.23Bd	424.53 ± 35.00Bab	586.08 ± 49.30Ab	2638.21 ± 182.33Bc	3354.50 ± 213.93ABc
N2	M1	2877.52 ± 350.34Abc	3359.93 ± 114.34Bbc	450.16 ± 97.13Aab	690.11 ± 28.75Ba	3327.68 ± 266.35Abc	4050.03 ± 97.82ABbc
	M2	2734.56 ± 426.40Ab	3253.58 ± 122.28BCab	373.56 ± 90.65Ab	661.58 ± 49.41Ba	3108.12 ± 442.55Ab	3915.15 ± 159.24ABab
	M3	2607.04 ± 346.76Ab	3048.82 ± 158.99Cc	470.66 ± 96.29Aab	788.42 ± 74.15Aa	3077.70 ± 269.39Aab	3837.23 ± 228.72Bc
	M4	2839.16 ± 141.96Abc	3636.78 ± 40.32Ab	351.82 ± 66.73Ab	531.87 ± 50.51Cb	3190.98 ± 132.29Abc	4168.65 ± 30.41Ab
N3	M1	3954.64 ± 426.18Aab	3739.67 ± 187.07Ba	533.32 ± 152.96Aa	591.40 ± 92.95Bb	2510.64 ± 77.57Aab	4331.07 ± 280.02Ba
	M2	4554.64 ± 271.78Aa	3322.67 ± 196.63Cab	578.47 ± 84.46Aa	484.53 ± 102.7Cb	4487.96 ± 382.97Aa	3807.19 ± 200.60Cb
	M3	3757.20 ± 73.28Bba	4077.81 ± 84.05Aa	295.91 ± 44.94Bbc	812.96 ± 36.38Aa	4053.11 ± 168.26Aa	4890.77 ± 80.85Aa
	M4	4254.96 ± 733.36Aa	4226.21 ± 78.57Aa	431.51 ± 38.95ABab	548.52 ± 63.53BCb	4686.46 ± 753.10Aa	4774.73 ± 99.78Aa
N4	M1	4153.28 ± 672.88Aa	3554.94 ± 80.98Bab	380.55 ± 120.25Bb	713.76 ± 23.46Aba	4533.83 ± 621.23Aa	4268.70 ± 89.11Bab
	M2	3297.28 ± 495.46Bb	3122.56 ± 78.08Cb	728.80 ± 61.58Aa	535.28 ± 22.88Cb	4026.08 ± 305.72ABb	3657.84 ± 89.43Cb
	M3	3041.36 ± 125.24Bab	3885.80 ± 151.63Aa	384.20 ± 73.82Bbc	800.04 ± 32.03Aa	3425.56 ± 23.51Bab	4685.84 ± 156.43Aa
	M4	3198.60 ± 112.00Bbc	3469.72 ± 78.16Bbc	579.04 ± 66.40Aa	677.98 ± 58.77Ba	3777.64 ± 121.68ABb	4147.69 ± 117.85Bb
N5	M1	3437.88 ± 144.92Aab	2868.68 ± 181.94Bd	379.18 ± 98.57Aab	422.24 ± 18.03Bc	3817.06 ± 229.26Aabc	3290.93 ± 177.76Bd
	M2	3322.20 ± 45.37ABb	3414.78 ± 98.86Aa	355.55 ± 104.80ABb	704.60 ± 44.68Aa	3677.75 ± 313.13Ab	4119.38 ± 143.18Aa
	M3	2576.56 ± 197.30ABb	3400.04 ± 171.78Ab	284.54 ± 52.67Bc	728.15 ± 39.31Aa	2861.1 ± 51.69Ab	4128.19 ± 207.00Ab
	M4	2774.24 ± 492.40Bbc	3351.83 ± 135.03Ac	453.50 ± 69.80ABab	674.73 ± 33.03Aa	3227.74 ± 184.14Abc	4026.56 ± 111.54Ab

Note: The capital letters indicated there was significantly difference at 0.05 levels under different planting density and same nitrogen fertilizer application, and lowercase letters indicated there was significantly difference at 0.05 levels under different nitrogen fertilizer and same planting density. The same below.

### The effect of planting densities and nitrogen fertilizer on contribution rate of Avena sativa L

#### Root contribution rate

Under N1 condition, the contribution rate of M3 was the biggest value in 2017 and 2018, and there was no different among densities treatments in 2017. Under N2 condition, the contribution rate of M4 was the biggest, and the value of M4 was significantly higher than M1 and M3 in 2017, and there was no different among densities treatment in 2018. Under N3 condition, there was no different among densities treatments in 2018 and M1 value was the biggest which remarkable higher than M3 in 2017. Under N4 condition, the root contribution rate of M2 and M4 was significantly higher than M1 and M3 in 2017, there was no different among densities treatments in 2018. Under N5 condition, there was no different among densities treatment in 2018, and M1 value was remarkable lower than other treatments in 2017 (Table 2).

**Table 2:** The effect of planting density and nitrogen fertilizer additions on each organs contribution ratio.

		Root mass ratio		Stem mass ratio		Leaf mass ratio		Panicle mass ratio	
		2017	2018	2017	2018	2017	2018	2017	2018
N1	M1	26.04 ± 6.82Aa	15.70 ± 1.19ABa	38.66 ± 5.51Aa	40.41 ± 0.57Aa	15.47 ± 3.30Aa	14.34 ± 0.47Ac	19.83 ± 4.10Ab	28.55 ± 2.24Aa
	M2	29.06 ± 7.90Aa	13.01 ± 1.12Ba	37.72 ± 6.37Aab	37.26 ± 3.29Ab	13.56 ± 3.70ABb	16.49 ± 1.79Abc	19.66 ± 7.09Ab	20.81 ± 2.48Bbc
	M3	30.70 ± 8.93Aa	19.19 ± 1.67Aa	38.68 ± 5.57Ab	37.45 ± 1.03Aa	11.85 ± 2.45Bc	12.02 ± 0.23Bb	18.76 ± 4.00Ac	29.08 ± 1.62Aa
	M4	25.51 ± 11.82Aab	15.72 ± 0.13ABab	38.14 ± 11.31Ab	42.15 ± 0.50Ab	13.79 ± 6.18ABa	15.16 ± 1.25Aa	22.56 ± 12.78Aa	22.52 ± 0.91Bb
N2	M1	17.33 ± 7.94BCb	17.86 ± 0.84Aa	41.03 ± 8.66Aba	42.15 ± 3.90Aa	16.48 ± 4.25Aa	17.45 ± 1.00Ab	25.17 ± 8.85Aa	22.54 ± 2.77ABc
	M2	23.35 ± 11.46ABab	15.85 ± 3.31Aa	36.97 ± 7.36Bb	40.98 ± 6.85Ab	17.26 ± 7.74Aa	17.15 ± 0.38Abc	22.42 ± 5.91Aab	24.52 ± 1.25Aab
	M3	14.97 ± 5.66Cc	19.09 ± 1.46Aa	44.02 ± 4.75Aa	41.69 ± 1.79Aa	17.72 ± 3.10Aa	13.55 ± 0.24Bb	23.29 ± 3.66Ab	19.10 ± 0.54Bc
	M4	24.91 ± 6.69Aab	19.20 ± 0.78Aa	38.52 ± 6.44Bb	43.40 ± 3.92Aab	13.59 ± 3.62Ba	15.42 ± 0.18Aba	22.97 ± 4.88Aa	22.88 ± 3.02ABb
N3	M1	26.34 ± 13.78Aa	16.45 ± 1.22Aa	40.44 ± 7.53Aa	42.97 ± 2.01Aa	13.94 ± 6.04Aa	18.13 ± 0.45Ab	19.28 ± 4.14Bb	24.11 ± 1.14ABbc
	M2	22.29 ± 13.40ABb	18.20 ± 1.77Aa	40.42 ± 7.60Aa	43.09 ± 1.01Aab	13.75 ± 3.41Ab	15.53 ± 0.22Bc	23.54 ± 4.23Aa	21.77 ± 1.29Bb
	M3	16.74 ± 7.34BCc	21.39 ± 3.63Aa	44.28 ± 7.37Aa	38.41 ± 3.32Aa	14.50 ± 3.20Abc	13.06 ± 0.29Cb	24.48 ± 3.61Aab	17.02 ± 0.70Cc
	M4	20.66 ± 10.44ABb	16.58 ± 1.18Aab	43.86 ± 6.99Aa	39.81 ± 1.17Ab	14.28 ± 2.35Aa	14.57 ± 0.69BCa	21.21 ± 4.48Aba	27.06 ± 1.26Aa
N4	M1	21.44 ± 7.92Bab	16.44 ± 0.51Aa	40.63 ± 7.92Aa	42.47 ± 0.28BCa	13.85 ± 2.78Aa	21.25 ± 2.05Aa	24.08 ± 4.86Aba	23.29 ± 1.09Ac
	M2	25.87 ± 10.90ABab	17.63 ± 2.92Aa	39.35 ± 5.98ABab	47.49 ± 0.50Aba	10.94 ± 4.89Bb	18.10 ± 0.52Bab	23.85 ± 4.32Aba	22.14 ± 0.07Ab
	M3	16.40 ± 8.55Cc	16.89 ± 0.68Aa	41.38 ± 9.37Aab	41.36 ± 2.15Ca	15.11 ± 3.25Aab	16.87 ± 1.27Ba	27.11 ± 4.13Aa	24.47 ± 0.75Ab
	M4	30.64 ± 8.28Aa	12.83 ± 2.61Ab	35.24 ± 4.96Bb	48.65 ± 1.79Aa	13.71 ± 2.60Aa	16.05 ± 0.68Ba	20.40 ± 6.03Ba	20.35 ± 0.08Ab
N5	M1	17.48 ± 10.14Bb	16.14 ± 2.64Aa	42.96 ± 5.81Aa	40.04 ± 3.30Aa	15.25 ± 5.08Aa	15.95 ± 1.37Bbc	24.31 ± 4.29Aba	27.86 ± 1.71Aab
	M2	25.78 ± 10.26Aab	14.33 ± 0.36Aa	35.03 ± 12.65Bab	43.37 ± 1.30Aab	13.28 ± 7.58Ab	19.58 ± 0.45Aa	25.91 ± 4.51Aa	27.08 ± 1.48Aa
	M3	23.74 ± 10.10Ab	18.36 ± 1.08Aa	40.20 ± 9.19Aab	37.88 ± 1.12Aa	13.63 ± 2.28Abc	16.25 ± 1.34Ba	22.43 ± 5.83ABb	26.91 ± 1.62Aab
	M4	26.33 ± 8.92Aab	19.26 ± 5.15Aa	38.51 ± 5.34ABb	40.02 ± 2.94Ab	13.54 ± 3.32Aa	16.74 ± 1.70Ba	21.62 ± 5.35Ba	23.99 ± 3.61Aab

Under M1 condition, the root contribution of N1 and N3 was remarkable higher than N2 and N5 in 2017, and no different among nitrogen fertilizer application in 2018. Under M2 condition, the value of N1 was significantly higher than N3 in 2017, and N1 was remarkable lower than other treatments in 2018. Under M3 condition, the value of N1 was significantly higher than N2, N3, N4 and N5, N5 remarkable higher than N2, N3 and N4 in 2017, and there was no different among treatments in 2018. Under M4 condition, N4 was significantly higher than N3 in 2017, and no different among treatments in 2018 (Table 2).

#### Stem contribution rate

Under N1 and N3 condition, there was no different among different densities treatments for stem contribution rate in 2017 and 2018. Under N2 condition, M3 was significantly higher than M2 and M4 in 2017, and no different among treatments in 2018. Under N4 condition, M1 and M3 were significantly higher than M4 in 2017, and M2 and M4 were remarkable higher than M1 and M3 in 2018. Under N5 condition, M1 and M3 were remarkable higher than M2 in 2017, and no different among treatment in 2018 (Table 2).

Under M1 condition, there were no significantly difference among treatments in 2017 and 2018. Under M2 condition, N3 was significantly higher than N5, with no significant difference among other treatments in 2017, and N4 was significantly higher than other nitrogen treatments in 2018. Under M3 condition, N1 was significantly lower than N2 and N3 in 2017, and there was no significant difference in nitrogen fertilizer treatments in 2018. Under M4 conditions, N3 was significantly lower than N1, N2, N4 and N5 in 2017, and N4 was significantly higher than other treatments in 2018 (Table 2).

#### leaf contribution rate

Under N1 condition, M1 was significantly higher than M3 in 2017, and M3 was significantly lower than other density treatments in 2018. Under the condition of N2, M4 in 2017 was significantly lower than N1, N2, N3 and N5, and M3 in 2018 was significantly lower than other density treatments. In 2017, there was no significant difference between M1, M2, M3 and M4 under N3 and N5 conditions. In 2018, M1 was significantly higher than other treatments under N3 treatment, and M2 was significantly higher than other treatments under N5 treatment. Under N4 conditions, M2 in 2017 was significantly lower than M1, M3 and M4, and M1 in 2018 was significantly higher than other treatments (Table 2).

Under M1 condition, there was no significantly difference among nitrogen fertilizer application treatments in 2017, and the leaf contribution rate of M4 was remarkable higher than other nitrogen fertilizer application treatments in 2018. Under M2 condition, the value of N1 was significantly higher than others in 2017, and N4 and N5 were remarkable higher than others in 2018. Under M3 condition, N2 was significantly higher than N1, N3 and N5, N1 was remarkable lower than N2 and N4 in 2017, and N4 and N5 were remarkable higher than others in 2018. Under M4 condition, there was no difference among nitrogen fertilizer application in 2017 and 2018 (Table 2).

#### Panicle contribution rate

In 2017, there were no significant differences among M1, M2, M3 and M4 under N1 and N2 conditions. The ear contribution rate of M4 was significantly lower than other treatments under N3 condition, and under N4 condition M4 was remarkable lower than M3, and M4 significantly lower than M2 under N5 condition. In 2018 there was no significantly different among treatments under N4 and N5 condition, M1 and M3 were significantly higher than M2 and M4 under N1 condition, and M3 was remarkable lower than others under N2 condition, and M4 was significantly higher than M2 and M3 under N3 condition (Table 2).

Under the condition of M1, N2, N4 and N5 in 2017 were significantly higher than that of N1 and N3, and in 2018, N1 was significantly higher than that of N2, N3 and N4. Under the M2 condition, N3, N4 and N5 were significantly higher than N1 in 2017, and N5 was significantly higher than N1, N3 and N4 in 2018. Under the condition of M3, N4 was significantly higher than N1, N2 and N5 in 2017, N1 was significantly lower than N2, N3, N4 and N5, and in 2018, N1 was significantly higher than N2, N3 and N4. Under M4 conditions, there was no significant difference between N1, N2, N3, N4 and N5 in 2017, and N3 was significantly higher than other treatments in 2018 (Table 2).

### The effect of planting densities and nitrogen fertilizer on root/shoot ratio and stem/leaf ratio of *Avena sativa* L

#### Root/shoot ratio

**Figure 2:**
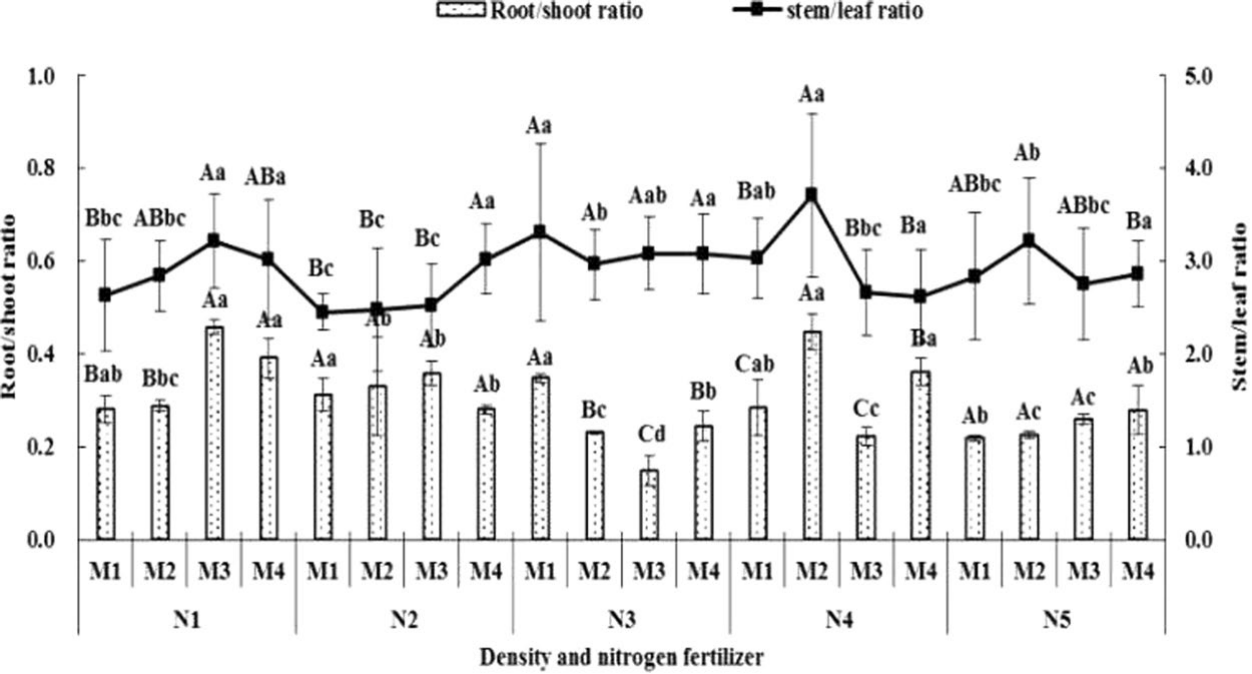
The effect of density and nitrogen fertilizer on root/shoot ratio and stem/leaf ratio of *Avena stative* L (2017).

**Figure 3:**
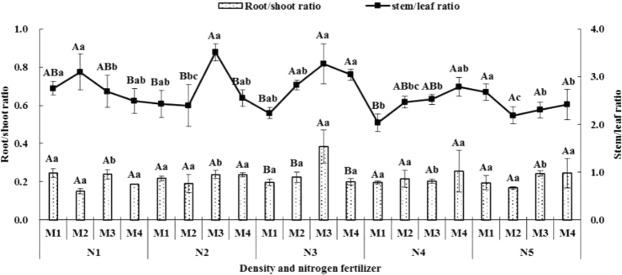
The effect of density and nitrogen fertilizer on root/shoot ratio and stem/leaf ratio of Avena stative L (2018).

Under same nitrogen fertilizer and different densities conditions, the root/shoot ratio indicated first increased and then decreased with rising densities under N1 and N2 conditions in 2017, and the biggest value appeared in M3, and the value of M3 and M4 was remarkable higher than M1 and M2 in N1 conditions, and there was no different among M1, M2, M3 and M3 in N2 conditions. The trend of root/shoot ratio showed first decreased and then increased under N3 conditions in 2017, and the lowest value appeared in M3, and which significantly lower than M1, M2 and M4, and the value of M1 was remarkable higher than M2, M3 and M4, and there was showed first increased and then decreased in 2018, and the value of M3 was the biggest. The root/shoot ratio of M2 was significantly higher than M1, M3 and M4 under N4 conditions in 2017. And the change trends of root/shoot ration showed gradually increased with rising densities under N5 conditions in 2017, and there was no different among M1, M2, M3 and M4 (Figs 2, 3).

Under M1 condition, the root/shoot ration value of N2 and N3 was significantly higher than N5 in 2017 and there was no different among different nitrogen fertilizers treatments in 2018. Under M2 condition, the value of N4 was remarkable higher than N1, N2, N3 and N5, and N2 higher than N1, N3 and N5 in 2017, and there was no different among nitrogen fertilizer treatments in 2018. Under M3 condition, there was significantly different among different nitrogen fertilizer treatments expect between N4 and N5 in 2017, and the change trend of root/shoot ration indicated first increased and then decreased with rising of nitrogen fertilizer treatment, and N3 was the biggest higher which remarkable higher than others in 2018. Under M4 condition, the value of N1 and N4 was significantly higher than N2, N3 and N5 in 2017, and there was no different among nitrogen fertilizer treatments in 2018 (Figs 2, 3).

#### Stem/leaf ratio

Under N1 condition, the change trend of stem/leaf ration showed first increased and then decreased with the rising of densities treatments, and the biggest value appeared M3 (2017) and M2 (2018). Under N2 condition, the change trend of stem/leaf ration indicated gradually increased with rising of densities treatment, and the value of M4 was significantly higher than M1, M2 and M3 in 2017, and first increased and then decreased with rising of densities treatment, and M3 was the biggest value in 2018. Under N3 condition, there was no different among M1, M2, M3 and M4 among different densities treatments in 2017 and first increased and then decreased with rising of densities treatments in 2018. Under N4 condition, it showed first increased and then decreased with rising of densities treatment in 2017 and gradually increased with rising of densities treatment in 2018. Under N5 condition, M2 was the biggest value, but there was no different among treatments in 2017, and first decreased and then increased with rising of densities in 2018 (Figs 2, 3).

Under M1 condition, the stem/leaf ratio value of N3 was significantly higher than N1, N2 and N5, and N2 was the lowest value which remarkable lower than N3 and N4 in 2017, and the change trend indicated first increased and then decreased with rising of nitrogen fertilizer application in 2018. Under M2 condition, N4 was the biggest value and which remarkable higher than others and N2 was the lowest which significantly lower than others in 2017, and in 2018 N5 was the lowest and remarkable lower than others. Under M3, N1 was the biggest value which was remarkable higher than N2, N4 and N5, and N2 was the lowest and remarkable lower than N1 and N3 in 2017, in 2018, the change trend indicated first increased and then decreased with rising of nitrogen fertilizers application in 2018. Under M4 condition, there was no different among treatments in 2017 and first increased and then decreased in 2018 (Figs 2, 3).

## Discussion

Nitrogen fertilizer is an important factor and which can affect the growth and development of Avena sativa L. The lach of nitrogen fertilizer application can cause leaves to lost chlorophyll, affecting photosynthesis and growth was restrained. Excessive nitrogen application will lead the stalk spindling and fall easily^15^. And studies have shown that the height of Avena sativa L. showed first increased and then decreased with the increase of the amount of nitrogen fertilizer application. It is shown that the application of appropriate nitrogen fertilizer can effectively improve the Avena sativa L. plant height, while the overdose has certain inhibitory effect. And the change rule is further verified in this experiment. And the biggest value of height was obtained under N3 nitrogen fertilizer application under same planting density. Planting density was the important factors on influencing the Avena sativa L. height, in the article, it showed that height of Avena sativa L. indicated increasing trend with the increase of planting density unding same nitrogen fertilizer application. And the result was similar with previous studies^16^. The main reason was that the coverage and density of *Avena sativa* L plant were improved and intensification of light competition by high planting density, and lead to the increase of the individual plants for obtaining more light. But there was no same change trend for biomass and height with increase of planting density, and higher biomass was obtained under lower planting density, such as M1 and M2. The reasons was that the yield of Avena sativa L was closely related to the number of tiller in addition to the high impact. And low density promoted the tillering of *Avena sativa* L, and high density restricted^17^.

Density and nitrogen fertilizer are important factors of yield for crops, and there are complementary between density and nitrogen fertilizer^17–19^. High density planting can reduce the effect of low nitrogen on oat yield, High nitrogen can increase the yield of Avena sativa L artificial grassland with low density. In this paper, the results also showed that there were no significant differences between different density Avena sativa L aboveground biomass under the condition of low nitrogen (N1 and N2), and high nitrogen (N3 and N4 interchange and N5) under the condition of low density (M1) planting Avena sativa L ground biomass is significantly higher than the density of planting (M4), and further suggests that under the condition of low density planting high nitrogen Avena sativa L biological effect higher than that of high density to increase production.

Under same planting density, aboveground biomass showed first increased and then decreased with nitrogen fertilizer application. The amount of nitrogen fertilizer applied to the highest aboveground biomass was slightly different in different densities, indicating that there was a certain exchange between density and nitrogen fertilizer. Under the condition of low nitrogen (N1 and N2), root-shoot ratio appears to increase with increasing planting density decreases after the change of the trend, perhaps the main cause is the low level of nitrogen in the soil nutrient content has become an important condition limit plant growth, plants depend on the increased amount of root system to get more chance to absorb nutrients. Therefore, the root crown ratio of m1-m3 was gradually increasing in low nitrogen condition. Under the condition of different density and nitrogen fertilizer into the quantity, in the process of data analysis and found no obvious change rule and trend, the reason may be due to certain interactions between nitrogen fertilizer and density effect.
